# Ensuring Continuity of Tuberculosis Care during Social Distancing through Integrated Active Case Finding at COVID-19 Vaccination Events in Vietnam: A Cohort Study

**DOI:** 10.3390/tropicalmed9010026

**Published:** 2024-01-22

**Authors:** Luong Van Dinh, Luan Nguyen Quang Vo, Anja Maria Christine Wiemers, Hoa Binh Nguyen, Hoa Quynh Vu, Huong Thi Lan Mo, Lan Phuong Nguyen, Nga Thi Thuy Nguyen, Thuy Thi Thu Dong, Khoa Tu Tran, Thi Minh Ha Dang, Lan Huu Nguyen, Anh Thu Pham, Andrew James Codlin, Rachel Jeanette Forse

**Affiliations:** 1 National Lung Hospital, Ha Noi 100000, Vietnam; dinhvanluong66@gmail.com (L.V.D.); nguyenbinhhoatb@yahoo.com (H.B.N.); hoavq118@gmail.com (H.Q.V.); 2 Friends for International Tuberculosis Relief (FIT), Ha Noi 100000, Vietnam; anja.wiemers@hotmail.com (A.M.C.W.); huong.mo@tbhelp.org (H.T.L.M.); nga.nguyen@tbhelp.org (N.T.T.N.); thuy.dong@tbhelp.org (T.T.T.D.); khoa.tran@tbhelp.org (K.T.T.); andrew.codlin@tbhelp.org (A.J.C.); rachel.forse@tbhelp.org (R.J.F.); 3 Department of Global Health, WHO Collaboration Centre on Tuberculosis and Social Medicine, Karolinska Institutet, 171 77 Stockholm, Sweden; 4 IRD VN Social Enterprise Company Limited, Ho Chi Minh City 700000, Vietnam; lan.nguyen@tbhelp.org; 5 Pham Ngoc Thach Hospital, Ho Chi Minh City 700000, Vietnam; hadtm2023@gmail.com (T.M.H.D.); nguyenhuulan1965@yahoo.com.vn (L.H.N.); 6 Hanoi Lung Hospital, Ha Noi 700000, Vietnam; drthuanh@gmail.com

**Keywords:** tuberculosis, COVID-19, active case finding, integrated service delivery, pandemic preparedness, Vietnam

## Abstract

COVID-19 significantly disrupted tuberculosis (TB) services in Vietnam. In response, the National TB Program (NTP) integrated TB screening using mobile chest X-rays into COVID-19 vaccination events. This prospective cohort study evaluated the integrated model’s yield, treatment outcomes, and costs. We further fitted regressions to identify risk factors and conduct interrupted time-series analyses in the study area, Vietnam’s eight economic regions, and at the national level. At 115 events, we conducted 48,758 X-ray screens and detected 174 individuals with TB. We linked 89.7% to care, while 92.9% successfully completed treatment. The mean costs per person diagnosed with TB was $547. TB risk factors included male sex (aOR = 6.44, *p* < 0.001), age of 45–59 years (aOR = 1.81, *p* = 0.006) and ≥60 years (aOR = 1.99, *p* = 0.002), a history of TB (aOR = 7.96, *p* < 0.001), prior exposure to TB (aOR = 3.90, *p* = 0.001), and symptomatic presentation (aOR = 2.75, *p* < 0.001). There was a significant decline in TB notifications during the Delta wave and significant increases immediately after lockdowns were lifted (IRR(γ_1_) = 5.00; 95%CI: (2.86, 8.73); *p* < 0.001) with a continuous upward trend thereafter (IRR(γ_2_) = 1.39; 95%CI: (1.22, 1.38); *p* < 0.001). Similar patterns were observed at the national level and in all regions but the northeast region. The NTP’s swift actions and policy decisions ensured continuity of care and led to the rapid recovery of TB notifications, which may serve as blueprint for future pandemics.

## 1. Introduction

Tuberculosis (TB) is a curable disease, yet 1-million to 2-million people die of TB annually, and the disease remains a major source of avoidable deaths worldwide [1]. The emergence of COVID-19 and the subsequent COVID-19 and TB syndemic had a devastating mortality impact. Studies estimate about 5.7-million deaths from both diseases at the height of the pandemic in 2021 [2]. Moreover, the associated mitigation measures at the global and national levels had deleterious consequences on TB care and prevention efforts [3,4]. The Stop TB Partnership estimates that the pandemic raised TB-related deaths [5] and reduced the number of persons with TB diagnosed and enrolled onto treatment to levels recorded in 2008 [6].

Vietnam had mounted a compelling public health response in the early stages of the pandemic. From national to community levels, the country managed to suppress each new outbreak [7]. This response included strong political commitment with the formation of a National Committee on COVID-19 led by the deputy prime minister, leading to clear messaging about social distancing, quarantining, and disallowing congregations. It also emphasized the need for washing hands and wearing face masks, rigorous reporting of travel history and potential exposure, rapid contact tracing and diligent record management [8]. Commune government enforcement of rapidly issued national guidance, coupled with neighborhood vigilance, resulted in fewer than 3000 reported infections and 35 deaths before May 2021 [9,10].

However, the highly transmissible Delta variant arrived in Vietnam in May 2021 and led to a rapid rise in infections and mortalities [11]. The government attempted to address this new wave of infections through the same strategies that proved effective during prior waves [12] in order to compensate for the delayed scale-up of vaccination campaigns [13]. However, as the death toll mounted, social distancing was replaced by societal lockdowns of increasing severity and scope [14,15].

When WHO declared the COVID-19 epidemic a public health emergency of international concern, it was clear that the immediate impact of the pandemic on daily activities would be severe. However, the aftermath of the aforementioned public health response to COVID-19 and deleterious impact on the country’s TB care and prevention system was less overt, thus necessitating further response to the initial public health response [16,17]. Specifically, the COVID-19 response exacerbated societal barriers to health-seeking, such as stigma and discrimination [18]. Additionally, accessing healthcare providers became increasingly challenging, especially at the primary and secondary levels and in the private sector. Fearing infection, quarantine, and forced closure of their businesses, many health facilities refused to examine people with respiratory symptoms [19,20,21]. Another challenge consisted of the repurposing of previous TB care capacity in terms of both facilities and human resources at the sub-provincial healthcare levels, i.e., district, commune and community, to serve COVID-19 control objectives, strongly impacting continuity of care [22,23]. The cumulative impact of lockdowns and access barriers was a substantial decline in TB notifications as treatment coverage declined to 46% compared to a baseline of 60% in 2019, resulting in 91,000 persons with TB unreached by Vietnam’s National TB Program (NTP) in 2021 [24,25].

To overcome the decline in notifications and to combat pandemic fatigue [26], the NTP and partners devised various strategies to reignite TB care and prevention efforts [27]. In alignment with WHO guidance, a large proportion of these strategies relied on greater community outreach and engagement [28]. Evidence on the effectiveness of a greater reliance on community outreach at the time had only begun to emerge [29], but despite the potentially lower yield and higher costs [30,31], the strategy of strengthening and engaging community health systems was rapidly adopted by major financing institutions such as the Global Fund’s COVID-19 Response Mechanism as a pillar for pandemic responses. One strategy rapidly deployed in Vietnam was to integrate mobile chest X-ray (CXR) screening for TB into COVID-19 vaccination activities [32]. The goal was to reduce access barriers to WHO-recommended molecular diagnostic tests for TB.

Here, we report on the results of the integrated screening campaigns. We also report local, regional, and national notification trends to highlight the strengths and limitations of the strategy in Vietnam’s effort to sustain its momentum towards achieving the country’s codified aspirations to end TB by 2030 [33].

## 2. Materials and Methods

### 2.1. Study Design, Aims, and Objectives

This was a prospective cohort study of a public health intervention with a complementary analysis of routine TB surveillance data. The aim was to assess the impact of integrated active TB case finding (ACF) at COVID-19 vaccination sites and trend changes on TB notifications in the intervention provinces, as well as at regional and national levels. Study objectives included: (1) describing the rates of TB detection, treatment initiation, and completion, and risk factors among people with TB detected through the integrated TB ACF intervention; (2) reporting of marginal TB screening and diagnosis costs; and (3) measuring changes in TB notification trends in the intervention provinces, as well as at regional and national levels before and after the Delta wave.

### 2.2. Study Setting

**Figure 1:**
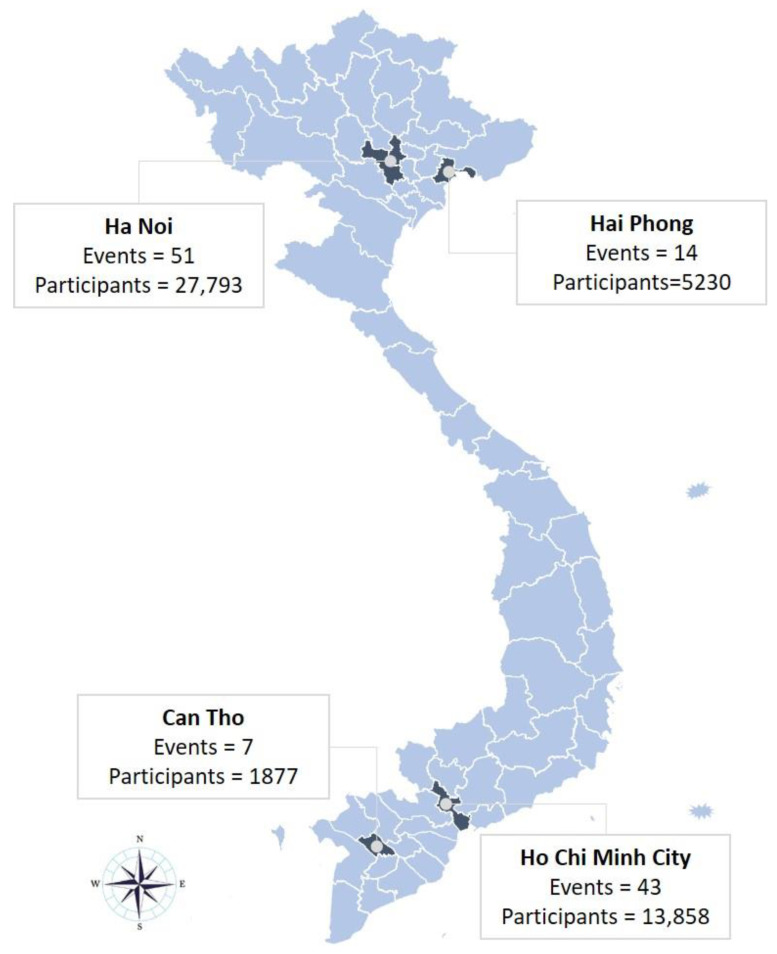
Map of screening events by study provinces.

At the height of Vietnam’s Delta wave between July 2021 and June 2022, 115 ACF events were held in selected districts of Ha Noi, Hai Phong, Ho Chi Minh City (HCMC), and Can Tho municipal provinces (Figure 1). The intervention provinces had a population of 4.6-million people and notified 5742 persons with drug-susceptible TB (DS-TB) in 2019. These provinces also represented epicenters of the Delta wave, with a cumulative 2.9-million individuals infected with COVID-19 and a related 22,313 mortalities by November 2023.

### 2.3. Eligible Study Population

The study population consisted of all persons attending government-organized COVID-19 vaccination events. People were eligible for TB screening if they were 18 years or older, did not have COVID-19, lived in the study area, and consented to participate. Persons contraindicated for CXR as per national TB treatment guidelines, e.g., pregnant women, were excluded.

### 2.4. Intervention

The intervention consisted of community-based mobile CXR screening events, which have been described in detail elsewhere [34]. Unlike previous campaigns, these screening activities occurred during social distancing and had to be integrated into vaccination events. To do so, a mobile X-ray van [35] or an ultra-portable radiography system [36,37] was established at a distance (e.g., 50–100 m) from the vaccine administration site and individuals in the post-vaccination waiting area were invited to participate to bridge the wait time. At vaccination sites with long pre-vaccination queues, individuals were also invited for TB screening before their COVID-19 vaccination. After providing informed consent, participants completed a verbal intake and the WHO 4-symptom screen of any duration (W4SS). All participants were invited for a CXR read by an on-site radiologist. Persons presenting parenchymal abnormalities suggestive of TB were invited to provide a sputum specimen for WHO-approved rapid molecular diagnostic testing. In both settings, sputum specimens were collected and transported to the nearest laboratory at the end of the day for testing. Treatment initiation and follow-up were provided by the NTP according to national TB treatment guidelines.

### 2.5. Data Sources

We used the NTP’s case-based community data-management system (Access to Care Information System [ACIS]; NTP, Ha Noi, Vietnam) for primary data collection. ACIS assigns a unique identifier to each participant and offers bespoke forms for demographic and clinical participant characteristics. Covariates included sex, age group, social health, insurance (SHI) enrollment, HIV co-infection, diabetes mellitus, exposure to any immunomodulatory drugs, congregate living conditions (e.g., prison, hospital, nursing home), history of TB, history of COVID-19, smoking, and positive W4SS for TB. CXR interpretations were batch-uploaded to ACIS at the end of each screening day. Sputum test results were obtained from NTP laboratory registers. District-level treatment registers furnished data on treatment linkage, outcomes, and all forms DS-TB notifications for the trend analysis in the intervention districts. National and regional notification data were available only on a quarterly basis and retrieved from the NTP’s surveillance system (VITIMES).

Cost data were obtained from the study’s accounting records. Types of costs included pre-event, during-the-event, and post-event follow-up expenses. Pre-event expenses included costs related to organizing planning meetings, printing, and photocopying, as well as renting X-ray equipment, tables, and fans. They also included the procurement of medical supplies, such as sputum collection tubes and large amounts of personal protective equipment. Costs during the event consisted largely of honoraria for supervisory staff and healthcare workers. Post-event expenditures consisted of fees for sputum collection and transport, consumables and labor for sample processing, stipends for clinical diagnosis, and transport for highly vulnerable groups. Costs were incurred in Vietnam Dong (VND) and converted into US Dollars (USD) using an exchange rate of USD 1 = VND 23,256 to represent the average spot rate during the study, per oanda.com.

### 2.6. Statistical Analyses

To assess the feasibility and yield of the integrated screening activities, we constructed a TB care cascade from persons screened to TB treatment completion. We calculated descriptive statistics of participant characteristics by TB diagnosis. Depending on the distribution and relevant assumptions of the parameter in question, we used Pearson’s chi squared test, Fisher’s exact test, and Wilcoxon rank-sum test to compare participant characteristics between cohorts of persons with and without TB. We calculated summary statistics for cost data and divided these by the number of persons screened and detected with TB to arrive at relevant marginal costs. We fitted a saturated, mixed-effect generalized logistic regression model to examine the relationship between positive TB diagnosis with demographic and clinical participant characteristics. The intraclass correlation coefficient was calculated to estimate clustering by province (ICC = 0.174, 95%CI: (0.044, 0.494)). Thus, the implementation province was specified as the random effect. Missing participant covariates were treated as a negative response. Persons without a sputum test result were excluded.

Trend changes due to the Delta wave were assessed by calculating incident rate ratios from single-group interrupted time-series analysis (ITSA) using segmented regression methods [38]. In the intervention area, we modeled pre-, intra-, and post-Delta periods with the monthly level data available. Interruptions between the segments were modeled in May and October 2021. May 2021 marked the beginning of the nationwide Delta wave in Viet Nam [39]. By the end of September 2021, the country reached sufficiently high levels of COVID-19 vaccination coverage for the government to issue Resolution No. 128 (128/NQ-CP 2021, dated 11 October 2021), which marked the shift from a strict “Zero-COVID” policy to a more flexible adaptation to the pandemic and the effective end of the Delta wave. As only quarterly data were available for analysis at the regional and national levels, we only modeled one interruption for the third quarter of 2021. For the regional disaggregation, we employed standard definitions of Vietnam’s eight economic regions. The ITSA employed a Poisson regression model using Generalized Estimating Equations (GEE). The Cumby–Huizinga test showed no autocorrelation in the monthly notification data for up to six lag periods, the length of the shortest segment. The test showed autocorrelation in the national quarterly data at two lag periods (*p* = 0.016), but there were insufficient observations to specify an autocorrelation structure, so an independent correlation structure was employed based on Quasi Information Criterion results. Model specification was assessed by visual inspection of residuals on the Q-Q plot. Meanwhile, *p*-values equal or less than 0.05 were considered significant. Data analysis was performed using Stata v17 (Stata Crop, College Station, TX, USA).

### 2.7. Ethical Considerations

The study was conducted in accordance with the Helsinki Declaration (7th Revision) and in strict compliance with guidelines and regulations of the Government of Vietnam. Ethical approval for study-specific data collection was granted by the Hai Phong University of Medicine and Pharmacy (01/HDDD, dated 8 February 2021) and the National Lung Hospital Institutional Review Board (12/21/CN-HDDD, dated 3 March 2021). Routine interventions were approved by the Ministry of Health (2528/QD-BYT, dated 16 June 2020; 3132/QD-BYT, dated 17 July 2020) and the HCMC People’s Committee (2681/QD-UBND, dated 29 July 2020; 3083/QD-UBND, dated 23 August 2021). All participants included in the analysis had provided informed consent. All data were anonymized prior to analysis.

## 3. Results

**Figure 2:**
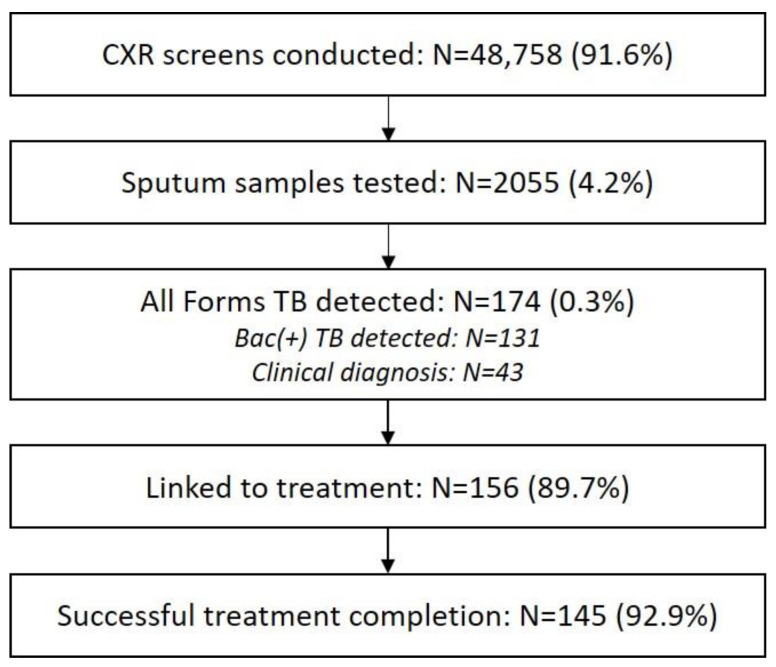
Aggregate TB care cascade of the integrated screening events.

In total, 48,758 participants were screened for TB by CXR (Figure 2), of whom 4.2% (2055/48,758) provided a sputum sample for testing, with a 6.4% (131/2055) positivity. Forty-three persons were clinically diagnosed for a total yield of 174 persons with TB. This corresponded to a TB detection rate of 357 per 100,000 persons screened and a number needed to screen (NNS) of 280. About 89.7% (156/174) of people with TB were initiated on treatment, and 92.9% (145/156) successfully completed their treatment.

Roughly 53.5% of participants were female (Table 1). The median age was 47 years with 30% between 45–59 and 23.4% above 60 years. About 74.2% had SHI coverage (74.2%), while 8.6% were confirmed smokers and 0.9% reported living or working in congregate settings. In terms of clinical characteristics, 8.6% had a concurrent or prior COVID-19 infection, 3.9% were confirmed diabetics, 0.3% were people living with HIV, and 0.7% indicated concomitant use of immunomodulatory drugs. Regarding TB-related covariates, 9.2% screened positive on the W4SS, 2.3% had a history of TB, and 1% reported known exposure to TB (see Univariate logistic regression of association between baseline characteristics and TB diagnosis in Table S1).

The multivariate regression showed an association between a TB diagnosis and male sex (aOR = 6.44; 95%CI: (4.21, 9.84); *p* < 0.001), age of 45–59 years (aOR = 1.81; 95%CI: (1.19, 2.75); *p* = 0.006) and ≥60 years (aOR = 1.99; 95%CI: (1.28, 3.10); *p* = 0.002), history of TB (aOR = 7.96; 95%CI: (5.27, 12.02); *p* < 0.001), prior exposure to a person with active TB disease (aOR = 3.90; 95%CI: (1.72, 8.84); *p* = 0.001), and presence of TB symptoms (aOR = 2.75; 95%CI: (1.86, 4.06); *p* < 0.001).

The average costs per person screened and diagnosed with TB were USD 1.78 and USD 547, respectively (Table 2). The cost per person detected was lowest in Can Tho (USD 191), due to the high TB detection rates in that province (see TB case detection and treatment cascade by site in Table S2).

**Figure 3:**
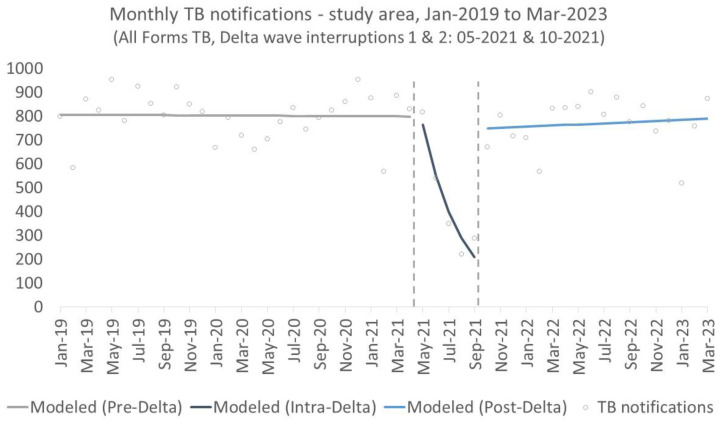
Interrupted time-series analysis of monthly TB notifications in the study area, January 2019 to March 2023 (Interruption 1: beginning of the Delta wave in May 2021; Interruption 2: relaxing of lockdown measures in October 2021).

With respect to monthly DS-TB notifications in the study area, there was no significant step change in notifications at the onset of the Delta wave (Figure 3 and Table 3), but there was a significant −28% decline in notifications during the lockdown period (IRR(β_2_) = 0.72; 95%CI: (0.64, 0.82); *p* < 0.001). Conversely, there was a significant step change (IRR(γ_1_) = 5.00; 95%CI: (2.86, 8.73); *p* < 0.001) and trend increase (IRR(γ_2_) = 1.39; 95%CI: (1.22, 1.38); *p* < 0.001) after the relaxation of social-distancing measures.

**Figure 4:**
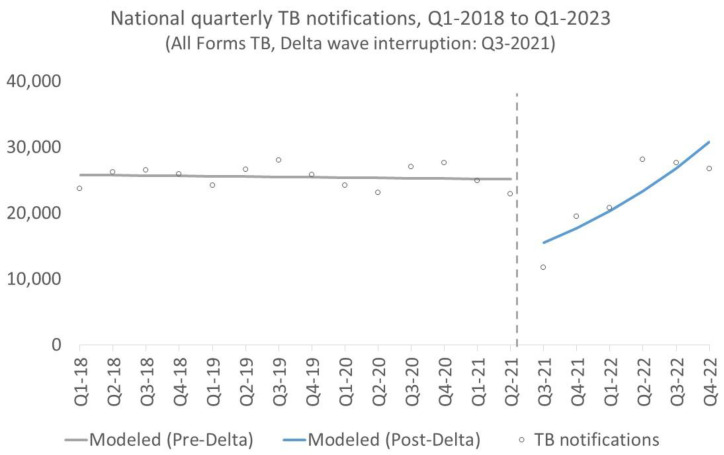
Interrupted time-series of national quarterly TB notifications, Q1-2018 to Q1-2023.

Quarterly national TB notifications (Figure 4 and Table 4) declined by −38% in 2021-Q3 (IRR(γ_1_) = 0.62; 95%CI: (0.47, 0.80); *p* < 0.001) and recovered at a rate of +15% per quarter thereafter (IRR(γ_2_) = 1.15; 95%CI: (1.07, 1.24); *p* < 0.001). At the regional level (Table 5), the decline in notifications in 2021-Q3 ranged from -15% in the northwest (IRR(γ_1_) = 0.85; 95%CI: (0.67, 1.07); *p* = 0.164) to −47% in the Mekong Delta (IRR(γ_1_) = 0.53; 95%CI: (0.38, 0. 67); *p* < 0.001). The declines in seven of eight regions were significant (0 < *p* ≤ 0.032), with the northwest being the notable exception. The average recovery rate of TB notifications ranged from +7% in the Red River Delta (IRR(γ_2_) = 1.07; 95%CI: (1.02, 1.13); *p* < 0.001) to +21% in the Mekong Delta (IRR(γ_2_) = 1.21; 95%CI: (1.09, 1.34); *p* < 0.001) per quarter. Recovery trends were significant across all regions (0 < *p* ≤ 0.025).

## 4. Discussion

Our study showed that the response mounted by the government of Vietnam and NTP in the aftermath of the Delta wave, including measures such as the integrated TB ACF events documented here, initiated the rapid recovery in TB case notifications both in the study area and across the nation. The concordance with prior studies of the TB cascade and risk factor analyses suggests that these ACF initiatives can indeed sustain access to care for vulnerable persons with TB and mitigate transmission even during social distancing, albeit with differences in the yield and costs. As yield was lower, i.e., the number needed to screen to detect a person with TB was higher, compared to events conducted prior to the pandemic, marginal costs per persons with TB detected were higher. This result was discordant with another study in Peru, where yields at integrated screening events were similar to the pre-COVID period. This was likely related to the target population, which consisted of symptomatic persons seeking care for COVID-19 in contrast to the general population in our setting [40]. Nevertheless, the long-term effect of initiatives like these by the NTP likely advanced the national health security agenda, and Vietnam’s pandemic preparedness and response.

At the individual level, the integrated screening campaign helped 48,758 persons receive CXR screening and TB diagnostic services during a time when access barriers were virtually insurmountable [41]. These efforts helped to identify 174 persons with TB among key vulnerable groups at risk of TB. This was substantiated by our risk factor analysis, which was concordant with prior evidence in terms of participant characteristics associated with TB, such as male gender, higher age, previous history of TB, prior exposure to a person with active TB, and symptomatic presentation [42,43]. In addition to reaching these vulnerable groups, our findings suggest that persons with TB were detected early in their disease course, as a high proportion (75.3%) reported no symptoms [44]. This rate was substantially higher than the 26–32% asymptomatic persons with TB documented during Vietnam’s two prevalence surveys [45,46]. Asymptomatic or subclinical TB is generally considered an early stage in the disease progression [47]. Detection of TB at this early stage in turn is associated with better treatment outcomes [48,49].

As early detection reduces transmission, this model of integrating TB screening into other health services possibly generated a positive public health impact [50], benefitting individuals in the same households [51], neighborhoods [52], and social circles [53]. A trial conducted in Vietnam on the basis of early detection systematically deployed community-wide ACF and demonstrated the ability to reduce TB prevalence in rapid fashion [54]. Thus, modeling exercises have repeatedly highlighted early detection of asymptomatic persons with TB, usually through ACF, as a key strategy for ending TB [55,56,57].

Conversely, the integrated screening strategy was characterized by a low yield of TB in relation to other ACF campaigns in Vietnam [53,58]. With an overall detection rate of 357 per 100,000 screened, yields were not substantially different from the prevalence survey result of 322 per 100,000 [46]. This was likely due to the indiscriminate mobilization of the population-wide vaccination campaigns, attracting many young, healthy individuals. A resulting disadvantage is the comparatively high cost per person with TB detected [34,59,60]. Despite the low yield, this model for integrated service delivery offered an advantage as well. Most of the study cohort was sampled from health-seeking individuals willingly presenting for COVID-19 vaccination. This high-value perception in one’s own health is reflected in the high rates of linkage to care and treatment completion, without much intervention or support after the screening events.

Beyond these individual and public health impacts, this model of integrated service delivery also may have served to strengthen the country’s pandemic preparedness and response by furnishing a real-life demonstration for sustaining essential medical services during public health emergencies. As COVID-19 outbreaks may become a chronic state of affairs with other pandemics looming, the lack of clear guidelines, processes, and mechanisms to sustain patient-centered TB care remains a key gap [61]. This gap exists in spite of the country’s commitment to the Global Health Security Agenda, as Vietnam only ranks 65/195 with a score of 42.91 on the Global Health Security Index [62]. Notable weaknesses include the capacity of clinics, hospitals, and community care centers, as well as socio-economic resilience and access to quality healthcare for communities vulnerable to biological threats. The latter was particularly exposed by the impact of the Delta variant, when existing inequalities facing informal workers, migrants, and other marginalized groups were highlighted and even exacerbated [63].

Conversely, the country has pledged its adherence to the International Health Regulations (IHR) (2005) and conducted regular Joint External Evaluations [64] and State Party Self-assessment Annual Reports [65] that have shown IHR coordination, communication and advocacy, and real-time surveillance as some of the key strengths. This is epitomized by the rapid communication by the NTP to all 63 provinces providing guidance on the integration of TB screening into COVID-19 vaccination campaigns (2297/BVPTW-DAPCL, dated 24 September 2021) issued 13 days after the first integrated screening event occurred at the height of the national lockdown. Hence, the integrated screening events symbolized the swift decisiveness exhibited by the government, as part of a greater cohesive strategy that characterized Vietnam’s strong public health response throughout the pandemic.

A key limitation of the study was related to the fears of local outbreaks resulting in a high degree of political uncertainty at the height of the Delta wave. This uncertainty meant consistent advocacy and follow-up with local leaders was required until permission was granted to implement integrated TB screening activities at COVID-19 vaccination sites. These permissions frequently arrived with less than a day’s notice, limiting the ability to mobilize the most vulnerable groups and requiring a high level of flexibility during the implementation. All these factors contributed to high heterogeneity in the procedures and data collection, thereby limiting the generalizability of our results. Heterogeneity also caused suboptimal levels of missing values in participant responses, particularly related to the history of COVID-19 and smoking. Nevertheless, for those individuals diagnosed with TB, these events represented one of the few opportunities to be diagnosed and treated. Thus, these limitations represented an acceptable compromise in exchange for sustained access to high-quality TB care.

## 5. Conclusions

The COVID-19 pandemic offered a glimpse of the devastating deleterious effects that pandemics can have on TB prevention and care. Thus, it is paramount for high TB burden countries to conduct further implementation research on methods for pandemic preparedness and response tailored to TB to develop strategies that can ensure a continuity of access to care for persons with TB. However, the pandemic also afforded the opportunity to develop strategies, processes, and capacities to ensure TB care systems are prepared and ready to respond appropriately. Our study showed that there are viable solutions for providing high-quality TB prevention and care services, even during times of strict social distancing and lockdowns, and that a country’s ambitions to end TB and to control an emerging pandemic are not mutually exclusive. More importantly, our study reiterated that it is critical to sustain the programmatic efforts, political commitment, and global momentum urgently needed to end TB by 2030.

## Figures and Tables

**Table 1:** Baseline characteristics and risk factors associated with a positive TB diagnosis.

	Total (N = 48,708)		TB (−) (N = 48,534)		TB (+) (N = 174)		aOR ^¶^	95% CI	*p*-Value ^§^
Sex									
Female	26,153	53.7%	26,127	54.0%	26	14.9%	Ref		
Male	22,555	46.3%	22,407	46.3%	148	85.1%	6.44	(4.21, 9.84)	<0.001
Age Group (N = 48,043)									
<15 years	499	1.0%	499	1.0%	0	0.0%	1	n/a ^¥^	n/a ^¥^
15–29 years	8973	18.7%	8959	18.5%	14	8.0%	0.79	(0.42, 1.49)	0.465
30–44 years	12,926	26.9%	12,892	26.7%	34	19.5%	Ref		
45–59 years	14,417	30.0%	14,350	29.7%	67	38.5%	1.81	(1.19, 2.75)	0.006
≥60 years	11,228	23.4%	11,169	23.1%	59	33.9%	1.99	(1.28, 3.10)	0.002
Median age (IQR)	47 (33–59)		47 (33–59)		53 (43–62)				
SHI coverage									
No	12,580	25.8%	12,544	25.9%	36	20.7%	Ref		
Yes	36,128	74.2%	35,990	74.4%	138	79.3%	0.98	(0.66, 1.44)	0.905
Smoking									
No/Unknown	47,481	97.5%	47,319	97.9%	162	93.1%	Ref		
Yes	1227	2.5%	1215	2.5%	12	6.9%	1.33	(0.69, 2.55)	0.390
Congregate living or working conditions									
No	48,259	99.1%	48,089	99.5%	170	97.7%	Ref		
Yes	449	0.9%	445	0.9%	4	1.7%	0.51	(0.10, 2.64)	0.423
HIV co-infection									
No	48,545	99.7%	48,374	100.0%	171	98.3%	Ref		
Yes	163	0.3%	160	0.3%	3	1.7%	0.34	(0.04, 3.25)	0.351
Diabetes mellitus									
No	46,830	96.1%	46,668	96.5%	162	93.1%	Ref		
Yes	1878	3.9%	1866	3.9%	12	6.9%	1.14	(0.57, 2.26)	0.712
Immunomodulatory treatment									
No	48,374	99.3%	48,205	99.7%	169	97.1%	Ref		
Yes	334	0.7%	329	0.7%	5	2.9%	3.30	(0.89, 12.17)	0.073
History of TB									
No	47,607	97.7%	47,467	98.2%	140	80.5%	Ref		
Yes	1101	2.3%	1067	2.2%	34	19.5%	7.96	(5.27, 12.02)	<0.001
Prior exposure to TB									
No	48,221	99.0%	48,057	99.4%	164	94.3%	Ref		
Yes	487	1.0%	477	1.0%	10	5.7%	3.90	(1.72, 8.84)	0.001
Any TB symptoms									
No	44,250	90.8%	44,119	91.2%	131	75.3%	Ref		
Yes	4458	9.2%	4415	9.1%	43	24.7%	2.75	(1.86, 4.06)	<0.001
COVID-19									
No/Unknown	44,520	91.4%	44,357	91.7%	163	93.7%	Ref		
Yes	4188	8.6%	4177	8.6%	11	6.3%	0.58	(0.29, 1.15)	0.121

Notes: ^¶^ Adjusted odds ratio based on the fitted multivariate regression model; ^§^ Wald Test; ^¥^ No individuals <15 years were diagnosed with TB.

**Table 2:** Cost per case detected and per person screened across the four provinces.

Province (Events)	Yield per 100,000	Cost per Person Screened by CXR (USD)	Cost per Person Diagnosed with TB (USD)
Total (*n* = 115)	325	1.78	547
Ha Noi (*n* = 51)	302	1.97	654
Hai Phong (*n* = 14)	200	0.64	322
Ho Chi Minh City (*n* = 43)	271	1.94	715
Can Tho (*n* = 7)	1758	3.36	191

Notes: CXR = Chest X-ray.

**Table 3:** Results of the interrupted time-series analysis of monthly TB notifications in the study area before, during, and after the Delta wave.

	IRR	95% CI	*p*-Value ^§^
Pre-Delta Trend (α_1_)	1.00	(0.99, 1.01)	0.916
Intra-Delta			
Step change (β_1_)	0.96	(0.81, 1.13)	0.614
Trend (β_2_)	0.72	(0.64, 0.82)	<0.001
Post-Delta			
Step change (γ_1_)	5.00	(2.86, 8.73)	<0.001
Trend (γ_2_)	1.39	(1.22, 1.58)	<0.001
Constant	805.65	(729.84, 889.35)	<0.001

Notes: ^§^ Wald Test; IRR = Incidence Rate Ratio based on Poisson regression employing Generalized Estimation Equations.

**Table 4:** Results of the interrupted time-series analysis of national quarterly all forms DS-TB notifications before and after the Delta wave.

	IRR	95% CI	*p*-Value ^§^
Pre-Delta Trend (α_1_)	1.00	(0.99, 1.01)	0.916
Post-Delta			
Step change (γ_1_)	0.62	(0.47, 0.80)	<0.001
Trend (γ_2_)	1.15	(1.07, 1.24)	<0.001
Constant	25,759	(24,338, 27,263)	<0.001

Notes: ^§^ Wald Test; IRR = Incidence Rate Ratio based on Poisson regression employing Generalized Estimation Equations.

**Table 5:** Results of the interrupted time-series analysis of quarterly TB notifications by economic region before and after the Delta wave.

	Red River Delta (IRR, 95%CI)	Northeast (IRR, 95%CI)	Northwest (IRR, 95%CI)	North Central Coast (IRR, 95%CI)	South Central Coast (IRR, 95%CI)	Central Highlands (IRR, 95%CI)	Southeast (IRR, 95%CI)	Mekong Delta (IRR, 95%CI)
Pre-Delta Trend (α_1_)	1.00 (0.99, 1.01)	0.99 (0.97, 1.00)	0.99 (0.97, 1.00)	0.98 (0.97, 1.00)	0.99 (0.98, 1.00)	1.00 (0.99, 1.02)	1.01 (1.00, 1.02)	1.00 (0.99, 1.00)
Post-Delta								
Step change (γ_1_)	0.62 (0.47, 0.80) *	0.73 (0.58, 0.93) ^	0.85 (0.67, 1.07)	0.58 (0.50, 0.66) *	0.66 (0.50, 0.89)	0.65 (0.51, 0.82) *	0.58 (0.35, 0.96) ^	0.53 (0.38, 0.76) *
Trend (γ_2_)	1.15 (1.07, 1.24) ^	1.10 (1.05, 1.16) *	1.08 (1.02, 1.15) +	1.19 (1.15, 1.23) *	1.14 (1.05, 1.24)	1.13 (1.06, 1.20) *	1.16 (1.02, 1.31) ^	1.21 (1.09, 1.34) *
Constant	4,834	2,035	306	2,292	2,031	693	6,978	6,618
	(4,430, 5,276)	(1,780, 2,326)	(280, 334)	(2,027, 2,590)	(1,865, 2,211)	(641, 750)	(6,669, 7,300)	(6,424, 6,817)

Notes: ^ = *p* < 0.05; + = *p* < 0.01; * = *p* < 0.001 by Wald Test; IRR = Incidence Rate Ratio based on Poisson regression employing Generalized Estimation Equations.

## Data Availability

The data that support the findings of this study are available from the Vietnam National Lung Hospital/NTP, Ha Noi Lung Hospital, Hai Phong Lung Hospital and Pham Ngoc Thach Hospital. However, restrictions apply to the availability of these data, which include programmatic clinical patient information, and so are not publicly available. Data can be made available from the authors upon reasonable request and with permission of the relevant government authorities listed above.

## Supplementary Materials

The following supporting information can be downloaded at: https://www.mdpi.com/article/10.3390/tropicalmed9010026/s1, Table S1: Univariate logistic regression of association between baseline characteristics and TB diagnosis. Table S2: TB case detection and treatment cascade by site.
