# Supplementary material for: Ensuring Continuity of Tuberculosis Care during Social Distancing through Integrated Active Case Finding at COVID-19 Vaccination Events in Vietnam: A Cohort Study
Source: Trop Med Infect Dis. 2024 Jan 22;9(1):26. doi: 10.3390/tropicalmed9010026 (PMC10819868; doi:10.3390/tropicalmed9010026)
Supplement: Supplementary file 1 [file tropicalmed-09-00026-s001.zip › tropicalmed-2786614-supplementary.pdf]

Supplementary Information

Supplementary results

Table S1. Univariate logistic regression of association between baseline characteristics and TB diagnosis.

Baseline Characteristics	OR	95% CI	P-value
Gender			
Female	Ref		
Male	6.58	[4.33- 9.98]	**<0.001**
Age Group (N = 48,043)			
<15 years	1	n/a	**n/a**
15-29 years	Ref		
30-44 years	0.59	[0.32- 1.10]	0.099
45-59 years	1.77	[1.17- 2.70]	**0.007**
≥60 years	1.99	[1.31- 3.05]	**0.001**
Social health insurance			
No	Ref		
Yes	1.33	[0.92- 1.91]	0.132
Co-infection with HIV			
No	Ref		
Yes	5.33	[1.69- 16.88]	**0.004**
Diabetes mellitus			
No	Ref		
Yes	1.86	[1.03- 3.35}	**0.038**
Altered immune system			
No	Ref		
Yes	4.36	[1.78- 10.68]	**0.001**
Congregate living or working conditions			
No	Ref		
Yes	2.56	[0.94 – 6.92]	0.065
Previous TB treatment			
No	Ref		
Yes	10.87	[7.43- 15.88]	**<0.001**
Contact person			
No	Ref		
Yes	6.15	[3.23- 11.72]	**<0.001**
Any TB symptoms			
No	Ref		
Yes	3.29	[2.33- 4.65]	**<0.001**
Smoking			
No	Ref		
Yes	2.90	[1.61-5.23]	**<0.001**

Covid-19			
No	Ref		
Yes	0.72	[0.39- 1.33]	0.294

Table S2. TB case detection and treatment cascade by site.

	Total	Hanoi	Ho Chi Minh City	Hai Phong	Can Tho
Screening locations	135	59	54	15	7
Days of screening	115	51	43	14	7
Screened by CXR	48,758	27,793	13,858	5,230	1,877
Abnormal CXR	2,051 (4.2%)	710 (2.6%)	938 (6.8%)	291 (5.6%)	112 (6.0%)
Sputum test after abnormal CXR	1,998 (97.4%)	710 (100.0%)	902 (96.2%)	274 (94.2%)	112 (100.0%)
Bac(+) TB detected after abnormal CXR	130 (6.5%)	65 (9.2%)	35 (3.9%)	13 (4.7%)	17 (15.2%)
Sputum testing outside of Double-X	57 (0.1%)	14	36	7	-
Bac(+) TB detected outside of Double-X	1 (1.8%)	1 (7.1%)	-	-	-
Clinically diagnosed TB	43 (0.1%)	18	3	6	16
All Forms of TB detected	174 (0.4%)	84	38	19	33
All Forms of TB linked to treatment	156 (89.7%)	76 (90.5%)	34 (89.5%)	17 (89.5%)	29 (87.9%)
All Forms of TB successfully treated*	145 (92.9%)	71 (93.4%)	29 (85.3%)	16 (94.1%)	29 (100.0%)
TB prevalence rate (per 100,000)	357	302	274	363	1,758

HN: Ha Noi; HCM: Ho Chi Minh City; HP: Hai Phong; CT: Can Tho; CXR: Chest X Ray; Double X: Rapid screening using CXR & GeneXpert; *cured/completed
